# A Surgical Case of Infected Popliteal Artery Aneurysm From Rare Bacteria, Eubacterium sp

**DOI:** 10.7759/cureus.55744

**Published:** 2024-03-07

**Authors:** Yuki Hayashi, Atsushi Harada, Keita Kamata, Naoki Eguchi, Masashi Tanaka

**Affiliations:** 1 The Department of Cardiovascular Surgery, Nihon University School of Medicine, Tokyo, JPN

**Keywords:** mycotic popliteal artery aneurysm, eubacterium bacteremia, infected popliteal artery aneurysm, eubacterium sp, popliteal artery aneurysm

## Abstract

A 79-year-old man presented to our hospital with complaints of a sudden worsening of swelling in the right popliteal fossa and fever persisting for a week. Upon close examination, an infected popliteal artery aneurysm (PAA) was identified. Given the risk of rupture, the patient was advised to undergo surgery. The surgical procedure involved resecting the infectious PAA using a lateral approach. Additionally, a bypass was performed from the superficial femoral artery to the below-knee artery, utilizing the great saphenous vein located at the posterior aspect of the knee. Surgical findings revealed a popliteal artery pseudoaneurysm. Preoperative blood cultures identified Eubacterium spp., and cultures of the inoperative aneurysm specimens confirmed the presence of the same bacteria. After surgery, inflammation quickly subsided, and the patient was discharged on postoperative day (POD) 41 after receiving transvenous antibiotic therapy. Although PAA accounts for approximately 80% of all peripheral arterial aneurysms, mycotic aneurysms are relatively rare. Eubacterium spp. is part of the human intestinal or oral flora, and very few reports of bacteremia have been published. The present case of bacteremia caused by Eubacterium sp. is very rare; to the best of our knowledge, no literature has been published on this topic.

## Introduction

Popliteal artery aneurysms (PAA) account for approximately 80% of peripheral aneurysms and often coexist bilaterally or with other aneurysms [1]. PAA is generally found in some patients with lower limb ischemia due to thromboembolism, and symptoms range from intermittent claudication to acute critical limb ischemia; however, rupture is rare, ranging from 0-7% [2]. Most of these are true aneurysms caused by degeneration, and those presenting as infected aneurysms are rare [3,4].

Bacteremia caused by Eubacterium sp. is extremely rare [5] and there have been no reports of infectious PAA. We present this valuable case, which was successfully treated through surgery.

## Case presentation

**Figure 1:**
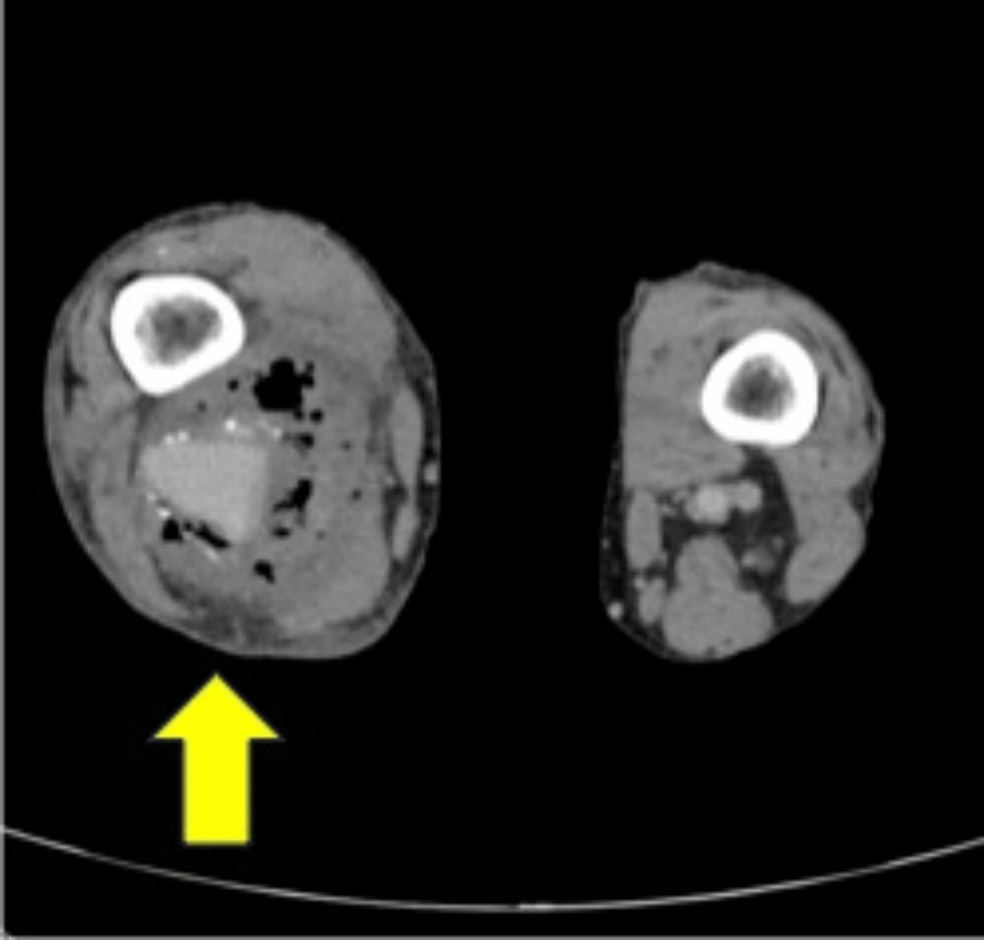
Preoperative horizontal computed tomography (CT) scan of the area around the PAA It shows a right popliteal aneurysm with free air in the surrounding tissue (arrow). PAA: popliteal artery aneurysm

**Figure 2:**
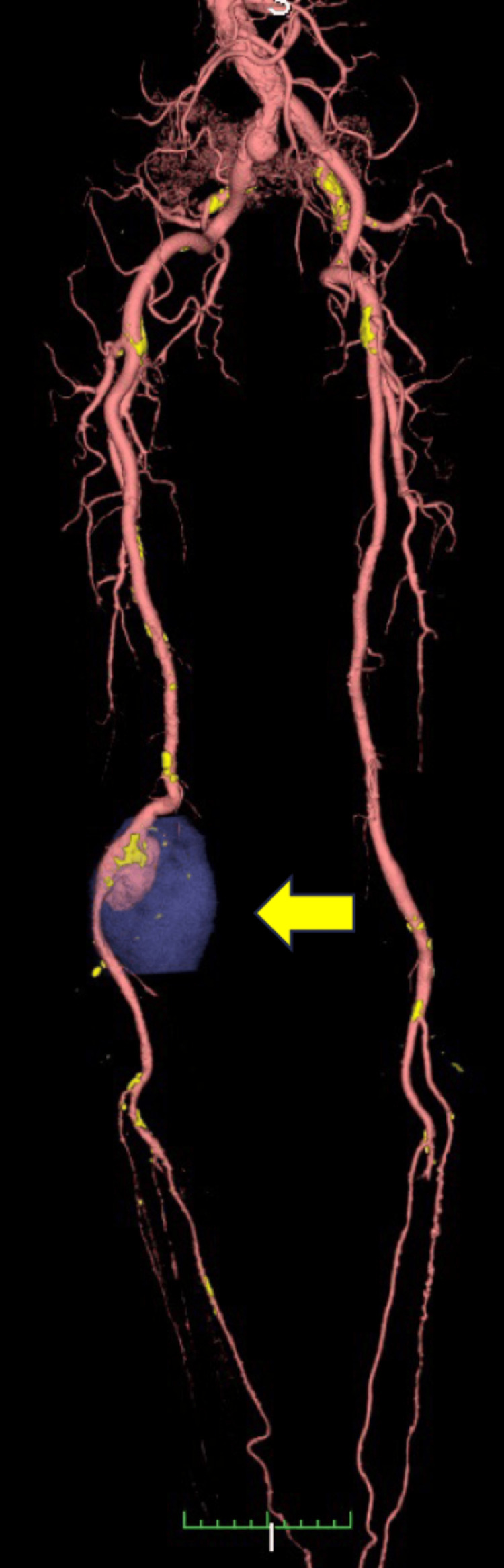
Preoperative 3D Computed tomography (CT) scan of arteries of the lower extremities This indicates that the aneurysm was saccular, with a maximum diameter of 34 mm × 24 mm (arrow), and that there was no blood flow into the aneurysm in part (blue-tined area).

A 79-year-old man, who had noticed swelling in the posterior right knee for one month, presented to the hospital with a chief complaint of worsening swelling and fever persisting for one week. The patient had a history of diabetes mellitus, and his glycated hemoglobin (HbA1c) level was 7.4%. On examination, the right knee was observed to be hot and swollen. Blood samples showed an elevated white blood cell count of 12800/μl and a C-reactive protein (CRP) of 14.13 mg/dl. Contrast-enhanced computed tomography revealed a right PAA with gas in the surrounding artery (Figures 1, 2).

**Figure 3:**
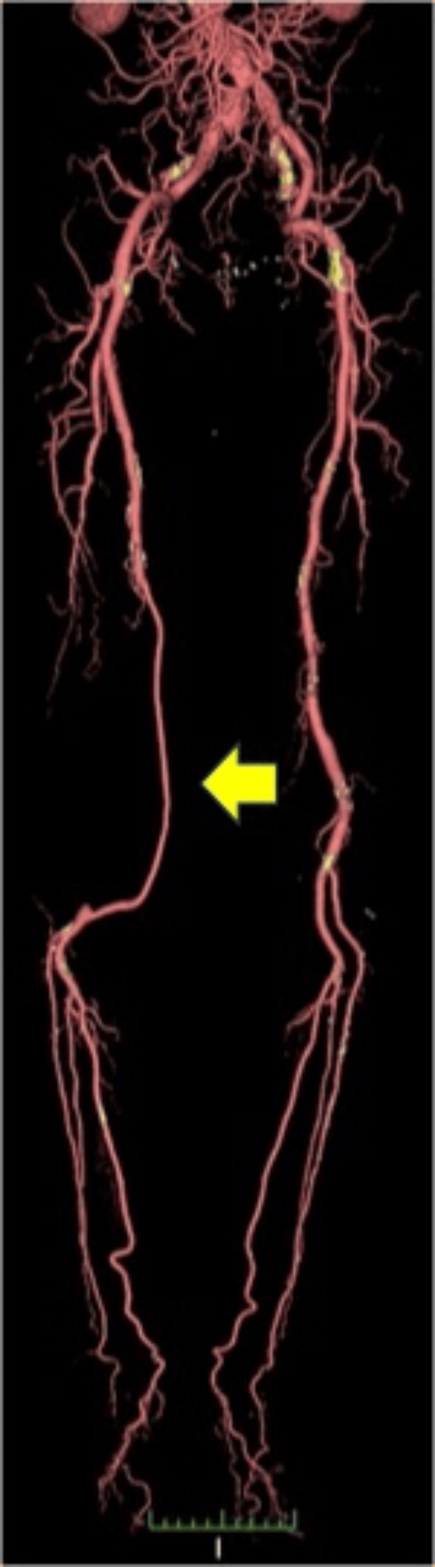
Postoperative three-year 3D computed tomography (CT) scan of arteries of the lower extremities This image shows that the bypass of the great saphenous vein flows well with no evidence of a recurrent aneurysm (arrow).

There were no abdominal infections such as intra-abdominal abscesses or obvious malignancies. Transthoracic echocardiography showed no evidence of infective endocarditis, and blood cultures detected Eubacterium sp. in two tubes. The results of these examinations led to a diagnosis of mycotic PAA. Surgery was scheduled following antibiotic administration. Initially, a superficial femoral-below popliteal artery bypass was performed using a great saphenous vein graft via the medial approach to the knee while the patient was in a supine position. After the wound was closed, we ligated and resected the mycotic PAA a posterior approach in the prone position. A large, dark red, viscous hematoma was observed around the aneurysm, which had partially ruptured and formed a pseudoaneurysm with no clear wall structure. The mass was removed as far as possible, the wound was thoroughly washed with saline solution, a drain was placed, and the wound was closed. The operative times were 4 h and 46 min, respectively. Intraoperative cultures of the aneurysm sac were positive for Eubacterium sp., similar to the preoperative blood cultures. After confirming a negative blood culture and improvement in the inflammatory response, the patient was discharged on the forty-first postoperative day. Three years after the surgery, there were no signs of infection, and the bypass was still open (Figure 3).

## Discussion

Infected PAAs are rare, accounting for approximately 1% of all PAAs, 50% of which are either ruptured or pseudoaneurysms [6]. The mean size of the ruptured PAA at presentation was 82 mm, and approximately 30% of these patients had an incidental abdominal aortic aneurysm, with approximately 62% having a contralateral PAA [7]. Streptococcus aureus and Escherichia (E.) coli are often detected as causative organisms of mycotic PAAs; however, Salmonella and Campylobacter have also been reported [3,8,9]. The routes of infection include infective endocarditis, gastroenteritis, pneumonia, infective abdominal aortic aneurysm, and urinary tract infection [4]. Mycotic PAAs pose a high risk of rupture and require prompt surgical treatment immediately upon diagnosis. The basic treatment strategy involves the removal of the aneurysm and infected tissue, followed by revascularization using autologous vein grafts [10]. There are two approaches for the treatment of infected PAA: medial and posterior. The former can be accessed above or below the popliteal artery and is easy to bypass while the latter can access the PAA directly and easily remove the infected tissue. Revascularization is typically performed in the second stage after achieving infection control, usually following the initial revascularization stage [11]. There have been some positive results with endovascular treatment [4], but we remain skeptical about recommending it for the removal of infected tissue and consideration of anatomical factors.

Eubacterium spp. are gram-positive anaerobic bacteria that have undergone repeated reclassification [12]. The majority of Eubacterium spp. inhabit the core of the enterobacterial flora in the human bowel and maintain a strong association with enteral health [13]. In addition, Eubacterium spp. may be identified in cases of intraoral pulp necrosis and periodontal disease, with diabetic patients being more susceptible to oral infections [14]. In patients with periodontal disease, plaque accumulation and gingival inflammation occur, and tooth brushing causes bacteremia [15].

The retrospective study by Bläckberg A et al. revealed that Eubacterium bacteremia is a highly uncommon form of bacteremia, with an incidence reported at 1.7 cases per million inhabitants per year [12]. Most of these bacteremia cases involved male patients with comorbidities such as a malignant tumor or an abdominal focus of infection, especially occurring in immunocompromised individuals. The mortality rates among these patients were low [12,16]. The study stated that 35% of Eubacterium bacteremia cases have an unknown source of infection. The detected separation stock often shows sensitivity to antibiotics, which are generally used in cases of abdominal infections in which an anaerobic bacterium is suspected [12].

The patient was a man with a history of diabetes mellitus. He did not have any immunodeficiency, and examination at the time of his visit revealed no abdominal infection such as a malignant tumor or an intra-abdominal abscess. The route of infection of the Eubacterium sp. bacteremia was unknown, but oral infections were not closely examined. Considering the patient's history of diabetes, it is highly likely that the patient was suffering from periodontal disease. As the surgical findings showed a pseudoaneurysm, it was thought that the existing PAA had rapidly expanded due to Eubacterium bacteremia, leading to rupture. To ensure the removal of the infected tissue, revascularization, and prevention of reinfection after surgery, we performed a one-stage surgery using medial and posterior approaches, and the patient's progress was good.

## Conclusions

In this report, we describe a case of infectious PAA caused by Eubacterium spp., which rarely causes bacteremia. Mycotic PAA requires surgical treatment, and it is important to save lives and limbs by using an appropriate therapeutic approach.
